# A PEDF‐Derived Short Peptide Prevents Sodium Iodate‐Induced Retinal Degeneration in Rats by Activating the SLC7A11/GSH/GPX4 Pathway in the RPE Cells

**DOI:** 10.1111/jcmm.70693

**Published:** 2025-07-24

**Authors:** Tsung‐Chuan Ho, Shawn‐H Tsai, Shu‐I Yeh, Ming‐Hui Sun, Yeou‐Ping Tsao

**Affiliations:** ^1^ Department of Medical Research Mackay Memorial Hospital New Taipei City Taiwan; ^2^ Department of Ophthalmology Mackay Memorial Hospital Taipei Taiwan; ^3^ Department of Medicine Mackay Medical College New Taipei City Taiwan; ^4^ Department of Optometry Mackay Medical College New Taipei City Taiwan; ^5^ Department of Ophthalmology Linkou Chang Gung Memorial Hospital Taoyuan Taiwan; ^6^ College of Medicine Chang Gung University Taoyuan Taiwan

**Keywords:** AMD, ferroptosis, PEDF, peptide drug, RPE, sodium iodate

## Abstract

Retinal pigment epithelial (RPE) cell damage caused by oxidative stress is a key factor in the pathogenesis of dry age‐related macular degeneration (AMD). 6dS peptide is derived from the neuroprotective motif of pigment epithelium‐derived factor (PEDF) and has antioxidant effects. This study used the sodium iodate (SI, a chemical oxidant)‐induced animal dry AMD model to investigate the 6dS‐mediated antioxidant mechanism. 6dS reduced SI‐induced cytotoxicity, including ferrous iron accumulation, lipid peroxidation, glutathione (GSH) depletion, and ferroptosis in ARPE‐19 cells. SI injection in rats induced cell death and lipid peroxidation in the RPE layer, along with retinal atrophy and electrophysiological dysfunction, recapitulating features of dry AMD that were counteracted by 6dS eye drop treatment. Mechanistically, 6dS induced the expression of SLC7A11 (solute carrier family seven member 11) and glutathione peroxidase 4 (GPX4) to alleviate SI‐induced GSH depletion and lipid peroxidation. Inhibitors targeting the PEDF receptor, SLC7A11, and GPX4 abolished the 6dS effect. Our study proposes an antioxidant mechanism through which PEDF receptor signalling links to the SLC7A11/GSH/GPX4 axis to alleviate intracellular redox imbalance. These findings suggest that 6dS eye drops may be a promising treatment for dry AMD.

## 1 Introduction

Dry AMD is the leading cause of irreversible vision impairment in individuals over 55 years old [1]. The pathophysiology of dry AMD is multifactorial, and oxidative stress‐related RPE injury is thought to be an early event in the development of AMD [2]. The RPE is responsible for supporting photoreceptor nutrition and degrading the tips of the photoreceptor outer segment (POS) to promote photoreceptor renewal [3]. RPE degeneration with age leads to incomplete POS degradation, leading to the progressive accumulation of lipofuscin, a source of reactive oxygen species (ROS), in RPE cells and promoting the development of geographic atrophic (dry) AMD [2, 3]. Treatment options for patients with dry AMD remain limited. In light of this, reducing RPE damage may be a key strategy to prevent or ameliorate the pathogenesis of dry AMD.

PEDF is a 50 kDa secreted glycoprotein produced by RPE cells, but its expression is reduced in aging RPE cells [4]. The RPE cells express the *PNPLA2* (patatin‐like phospholipase domain‐containing 2) gene, which encodes the PEDF receptor (PEDFR) and exhibits phospholipase A2 (PLA2) activity [5]. Conditional knockout of *PNPLA2* in mouse RPE cells has been shown to severely impair POS digestion [6]. PNPLA2 is also termed adipose triglyceride lipase (ATGL), and its PLA2 activity is blocked by the inhibitor atglistatin, which serves as a tool compound to study the pathophysiological role of ATGL in animal disease models [7, 8]. PEDF is regarded as a protective factor for retinal and RPE cells under oxidative stress conditions [9, 10, 11]. Recently, we identified a short neuroprotective fragment in PEDF (6‐mer; Ser93‐Gln98) and demonstrated that the d‐form 6‐mer variant (6dS) can reduce the toxicity of tert‐butyl hydroperoxide in 661W cells [10]. However, its molecular mechanism is still unclear.

Ferroptosis is implicated in the pathological process of oxidative stress‐mediated RPE degeneration [12, 13, 14, 15]. Ferroptosis is a regulated cell death process associated with the accumulation of intracellular labile iron ions (Fe^2+^), which accelerates the production of lipid peroxides, leading to cell membrane damage [16]. GPX4 is the only mammalian enzyme capable of catalysing phospholipid hydroperoxides to phospholipid alcohols, thus protecting RPE cells from ferroptosis [13, 17]. The reactivation of GPX4 is primarily supported by the system Xc^−^ (cystine/glutamate antiporter)/glutathione (GSH) axis [18]. SLC7A11 is a subunit of the system Xc^−^, responsible for transporting extracellular cystine into the cell to synthesise the antioxidant GSH [18]. In a mouse model of dry AMD induced by sodium iodate (SI), intravitreal injection of PEDF‐expressing adenovirus ameliorates SI‐induced retinopathy [15]. Furthermore, PEDF upregulates the expression of GPX4 and ferritin heavy chain 1 (FTH1; a Fe^2+^ storage protein) in RPE cells [15]. These suggest that activation of ferroptosis negative regulation (FNR) is one of the PEDF‐mediated antioxidant mechanism, although the exact mechanism by which PEDF regulates GPX4 activity remains unclear.

In this study, we explored the potential protective effect of 6dS on cultured ARPE‐19 cells and rat RPE cells challenged with SI. Our results showed that 6dS suppressed SI‐induced RPE ferroptosis, suggesting that it can recapitulate the FNR activity of PEDF. In animals, our results showed that topical application of 6dS eye drops was a simple and effective treatment for RPE and retinal damage caused by SI challenge. We also found that 6dS reduced SI‐induced GSH depletion by inducing the expression of SLC7A11 in ARPE‐19 cells. Our findings propose an antioxidant mechanism through which PEDFR signalling connects to the system Xc^−^, contributing to GSH metabolism and mitigating intracellular redox imbalance.

## 2 Materials and Methods

### 2.1 Materials

GPX4, FTH1 and SLC7A11 antibodies were obtained from Cell Signalling Technology (Danvers, MA, USA). Atglistatin, ML162 and HG106 were sourced from Selleckchem (Houston, TX, USA). Sodium iodate (NaIO_3_; S4007), dimethyl sulfoxide (DMSO) and haematoxylin and eosin (H&E) were acquired from Sigma‐Aldrich (St. Louis, MO). The 6dS peptide and 6dE control peptide (Cont‐P) [10] were synthesised by GenScript (Piscataway, NJ), and each peptide was modified by acetylation at the NH_2_‐terminus and amidation at the COOH‐terminus for stability and characterised by mass spectrometry (> 95% purity). The peptide was dissolved in DMSO to prepare a 10 mM stock, according to the peptide service recommendations.

### 2.2 Cell Culture and 6dS Treatment

ARPE‐19 cells (2.5 × 10^5^) were seeded in one well of a six‐well culture plate and cultured in Dulbecco's modified Eagle's medium (DMEM)/F12 medium with 10% FBS and antibiotics (100 U/mL penicillin, 100 μg/mL streptomycin) at 37°C in 5% CO_2_. After 24 h, cells that reached near confluence were switched to 2% FBS DMEM/F‐12 medium and treated with 20 μM 6dS or 0.2% DMSO solvent for 6 h, and then, SI was directly added to the culture medium.

### 2.3 Assessment of Cell Viability

**FIGURE 1:**
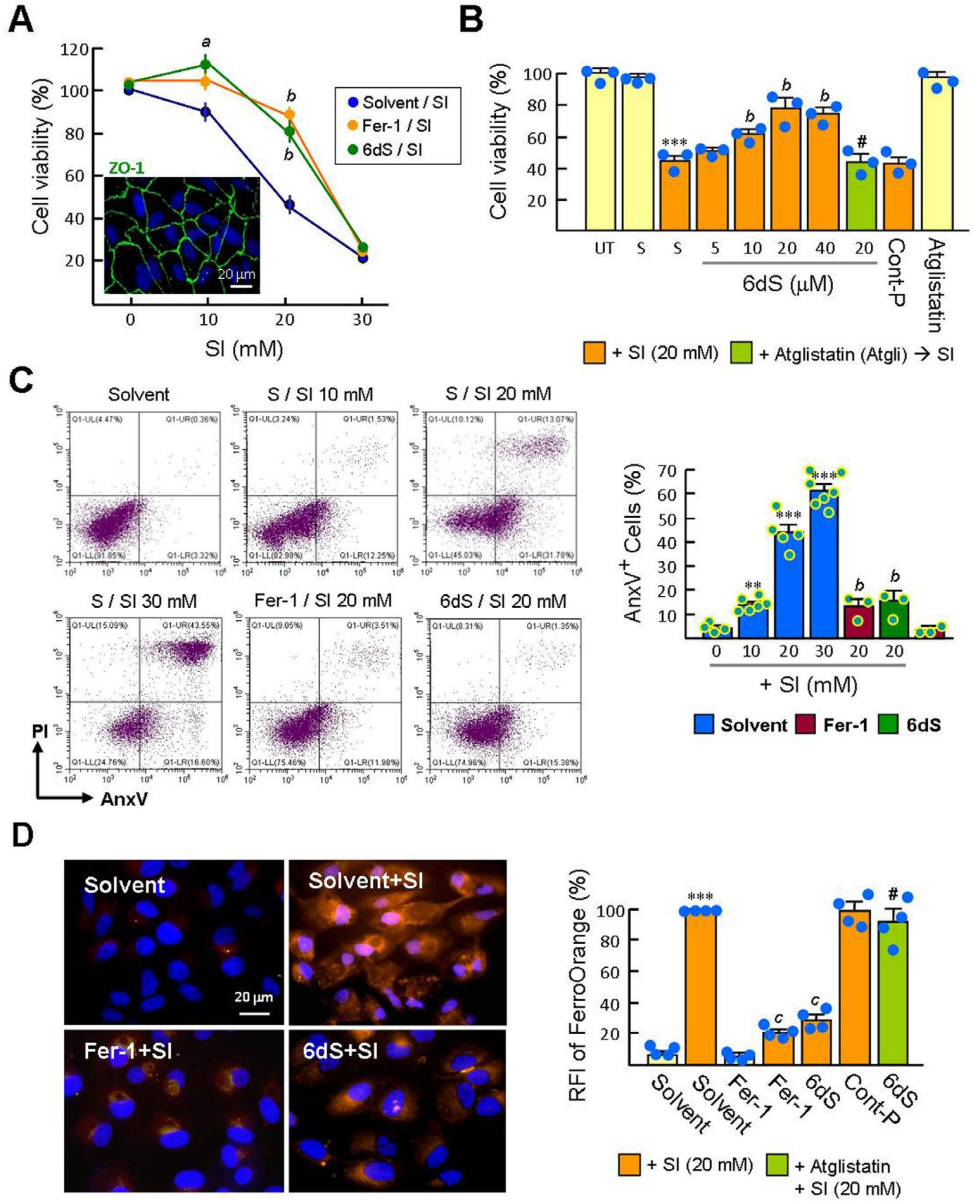
Pretreatment with 6dS reduces SI‐induced ferrous ion accumulation and cell death in ARPE‐19 cells. Tight junctions expressed in nearly confluent ARPE‐19 cells were monitored by ZO‐1 immunostaining. Subsequently, cells were pretreated with 20 μM 6dS, 6dS solvent (DMSO) or 20 μM Fer‐1 for 6 h, followed by SI treatment (10–30 mM) for 16 h. To inhibit PEDFR activity, cells were exposed to 20 μM atglistatin for 20 min prior to 6dS treatment. (A and B) Cell viability measured by CCK‐8 assay (*n* = 3). UT, Untreated cells. (C) SI cytotoxicity assessed by AnxV‐FITC/PI double staining and flow cytometry. The percentage of AnxV‐positive cells (UR + LR) is presented in a bar graph. (D) Representative fluorescence images of intracellular ferrous ions stained with FerroOrange and corresponding relative fluorescence intensity (RFI) analysed using Image Pro software. Data are presented as means ± SEM, **p < 0.01, and ***p < 0.001 versus solvent‐treated cells. ^a^ *p* < 0.05, ^b^ *p* < 0.01, and ^c^ *p* < 0.001 versus solvent/SI‐treated cells. ^*#*^ *p* < 0.01 versus 6dS/SI‐treated cells.

The viability of ARPE‐19 cells was evaluated using Cell Counting Kit‐8 (CCK‐8; Enzo Life Sciences, Farmingdale, NY, USA) according to the manufacturer's protocol. 5 × 10^3^ cells were seeded into the wells of a 96‐well culture plate for 24 h and then treated as described in the legend of Figure 1. CCK‐8 solution (10 μL/well) was added for 2 h, and then, absorbance was measured at 450 nm using a microplate reader.

### 2.4 Detection of Intracellular Ferrous Ions

Ferrous ion levels in viable cells were detected using 1 μM FerroOrange (EMD Millipore Corporation, Temecula, CA, USA) in 500 μL of balanced salt solution (BSS; Alcon Laboratories, Texas, USA) and stained for 30 min at 37°C. After three washes, images were captured using a Zeiss epifluorescence microscope (Zeiss Axioplan 2 Imaging; Zeiss, Oberkochen, German), equipped with a CCD camera (Zeiss AxioCam HRm).

### 2.5 Assessment of AnxV‐ and Acrolein Positive Cells

After 16 h of SI treatment, cells were stained using the Annexin V‐FITC Apoptosis Detection kit (BioLegend, California, USA). The percentage of AnxV‐positive cells was evaluated using flow cytometry (FACScaliber; Beckman Coulter, Brea, CA). In addition, cells were stained with AnxV‐FITC (5 μL per 1 × 10^6^ cells) for 15 min on ice, followed by fixation in 4% paraformaldehyde (PFA) and blocking with 10% goat serum and 5% bovine serum albumin (BSA) in phosphate‐buffered saline (PBS) for 15 min. Cells were then stained with acrolein primary antibody (1:250 dilution, ab240918; Abcam, Cambridge, UK) for 1.5 h at room temperature 37°C, followed by appropriate rhodamine‐conjugated donkey IgG (1:500 dilution) for 1 h at room temperature (RT). Images were captured using a Zeiss epifluorescence microscope.

### 2.6 Measurement of Intracellular ROS

After 4 h of SI stimulation, cells were incubated with medium containing 2.5 μM 2′,7′‐dichlorodihydrofluorescein diacetate (H_2_DCFDA; Thermo Fisher, MO, USA) for 15 min in the dark at 37°C. H_2_DCFDA is oxidised by ROS to form the green fluorescent compound 2′,7′‐dichlorofluorescein (DCF). DCF fluorescence (excitation/emission = 488 nm/520 nm) was measured using a SpectraMAX GEMINI Reader (Molecular Devices, Sunnyvale, CA).

### 2.7 Detection of Malondialdehyde (MDA) and GSH Levels

MDA levels were measured using the Lipid Peroxidation (MDA) Assay kit (ab118970; Abcam) according to the manufacturer's protocol. MDA in the sample reacts with thiobarbituric acid (TBA) to form an MDA‐TBA adduct, with absorbance measured at 532 nm.

Total glutathione (GSH+GSSG) and reduced GSH concentrations were assessed using colorimetric assay kits (ab239709; Abcam). For total GSH measurement, GSH reductase was added to convert GSSG to GSH, reacting with 5,5′‐dithiobis(2‐nitrobenzoic acid) to form a yellow product, measured at 412 nm. GSH reductase was omitted to detect reduced GSH. Protein concentration was determined with the Bio‐Rad Protein Assay (Bio‐Rad Laboratories, Hercules, CA, USA).

### 2.8 Semi‐Quantitative Real‐Time PCR

Total RNA was extracted from ARPE‐19 cells to synthesise cDNA as previously described [19]. The primers used in the experiment are listed in Table 1. The expression level of the β‐actin gene (*Actb*) as the internal reference gene was judged through the slight differences in the quantification cycle (Cq) and standard deviation (SD) between the experimental groups. All determinations were performed in triplicate, and the fold change of interest mRNAs were normalised to *Actb* using the $2^{-\Delta \Delta C_{t}}$ method.

**TABLE 1:** Primers used for real‐time qPCR.

Target gene	Primer (sense)	Primer (antisense)	Accession no.
hSLC7A11	5′‐CCTGACCAACATGGAGAAAC	5′‐CACACACACACACACACAC	NM_014331.4
hGPX4	5′‐CCCGACAGGTGATAGAGAAG	5′‐ATTCCCACAAGGTAGCCAG	AY324108.1
hFTH1	5′‐TACGCCTCCTACGTTTACC	5′‐CTCTCCCAGTCATCACAGTC	NM_002032.3
hACTB	5′‐TGCCATCCTAAAAGCCACC	5′‐ACCAAAAGCCTTCATACATCTC	NM_001101.5

### 2.9 Western Blot Analysis

Cell lysates were separated on 12% SDS‐polyacrylamide gel electrophoresis (PAGE) and then electrotransferred to polyvinylidene fluoride (PVDF) membranes (Immobilon‐P; Millipore) as previously described [19]. Antibodies against GPX4, FTH1, and SLC7A11 (1:1000 dilution) were used. Band intensities in immunoblots were measured using a Model GS‐700 imaging densitometer (Bio‐Rad Laboratories) and analysed using Labworks 4.0 software.

### 2.10 Animal Studies

Standard laboratory chow and tap water were available ad libitum. Experimental procedures were approved by the Mackay Memorial Hospital Review Board (project code: MMH‐A‐S‐110‐50; New Taipei City, Taiwan). Animals in ophthalmic studies were treated in compliance with the ARVO Statement and the ARRIVE Guidelines. Adult male Sprague‐Dawley rats (10 weeks old; initial body weight = 312 ± 11 g) were supplied by BioLASCO (Taiwan) and anaesthetized by an intraperitoneal injection of a mixture of ketamine (20 mg/kg body weight) and xylazine (10 mg/kg). All animals were euthanized by CO_2_ inhalation prior to dissection.

### 2.11 Rat Model of Retinal Degeneration and 6dS Eye Drop Treatment

SI was dissolved in BSS to achieve a 4% (w/v) concentration. Rats received a single intraperitoneal injection of freshly prepared SI at a dose of 40 mg/kg to induce RPE cell damage. 6dS and its solvent DMSO were dissolved in BSS to prepare 1 mM 6dS and vehicle (1% DMSO) eye drops for topical treatment as previously reported [10]. For subconjunctival injection of inhibitors, rats were anaesthetised and the eyes were briefly cleaned with 0.5% povidone‐iodine, and then, 0.1 mL of 200 μM inhibitor or DMSO solvent was injected into the subconjunctival space before topical 6dS eye drop treatment.

### 2.12 Immunofluorescence Staining

Eyes were fixed in 4% PFA overnight. Deparaffinised retinal sections were blocked with 10% goat serum and 5% BSA in PBS containing 0.5% Triton X‐100 (PBST) for 20 min at RT to block nonspecific staining. Staining was performed using primary antibodies against PEDF (1:100 dilution), RPE65 (1:100 dilution) or ZO‐1 (1:100 dilution; all from Abcam) for 3 h at RT 37°C. Slides were then incubated with appropriate fluorescently labelled secondary antibodies (1:500 dilution) for 1 h at RT 37°C and counterstained with Hoechst 33258 for 7 min. Slides were then rinsed three times with PBST, mounted with FluorSave reagent (Calbiochem), and observed using a Zeiss epifluorescence microscope.

### 2.13 TUNEL Staining

Retinal sections (5 μm) were deparaffinised, rehydrated, and incubated with 20 μg/mL proteinase K for 15 min. TUNEL (terminal deoxynucleotidyl transferase dUTP nick end labeling) staining was performed using the In Situ Cell Death Detection Kit (Roche Molecular Biochemicals, Indianapolis, IN) according to the manufacturer's instructions. Nuclei were counterstained with Hoechst 33258 for 7 min. Sections were observed under a Zeiss epifluorescence microscope. Three sections from each eye were photographed and used to count the number of TUNEL‐positive cells in the RPE.

### 2.14 Fundus Photography

Fundus images were obtained 7 days after SI injection using a Micron III retinal‐imaging microscope for rodents (Phoenix Research Labs, Pleasanton, CA, USA) equipped with a CCD camera.

### 2.15 Histological Evaluation of SI‐Induced Retinal Injury

Rats (*n* = 6 in each group) were euthanized 21 days after SI injection, the eyeballs were marked at the 12 o'clock position of the cornea with silk suture, and then, the eyes were enucleated and fixed in Davidson Solution (A3200; AppliChem Panreac) for 2 h at RT. After fixation, the anterior segment was removed, and the posterior eyeball containing the optic disc was dehydrated in a graded ethanol series and embedded in paraffin. For H&E staining, 5‐μm‐thick sections were taken along the vertical meridian of the optic nerve head using a microtome and observed at 200× under an optical microscope (Leica, Heidelberg, Germany) equipped with a CCD camera. For each section, photographs were taken 2000 μm from the optic nerve head, and the average thickness of the inner nuclear layer (INL) and outer nuclear layer (ONL) was measured at 500 μm distance intervals.

### 2.16 Scotopic Electroretinography (ERG)

The retinal ERG response of SD rats was detected as previously described [10]. Briefly, rats were dark adapted overnight and then anaesthetised. The pupils were dilated in dim red light with 2.5% phenylephrine hydrochloride ophthalmic solution (Akorn Laboratories). ERG was recorded using an Electrodiagnostic System (BPM‐300; RetinoGraphics Inc., CT, USA). ERG was detected using a stainless steel wire loop (0.1 mm diameter) contacting the corneal surface with 2.5% hypromellose ophthalmic demulcent solution (Akorn). The ERG was obtained by averaging the responses to two flashes of 2.5cd s/m^2^ (0dB) 2 min apart.

### 2.17 Statistical Analysis

Data were generated from at least three independent experiments. TUNEL assay results were compared using the Mann‐Whitney *U* test. The statistical significance of differences between groups was examined using one‐way analysis of variation (ANOVA), followed by Dunnett's post hoc test. All values are expressed as mean ± standard error of the mean (SEM). *p* < 0.05 was considered significant.

## 3 Results

### 3.1 6dS Alleviates SI‐Induced Ferroptosis in ARPE‐19 Cells

SI cytotoxicity in ARPE‐19 cells was assessed using the CCK‐8 assay. A significant decrease in cell viability was observed as the SI concentration increased from 10 to 30 mM (Figure 1A). Pretreatment with the ferroptosis inhibitor ferrostatin‐1 (Fer‐1) mitigated the decrease in cell viability induced by 10 and 20 mM SI (103.9% ± 4.5% and 88.8% ± 4.7% versus 87.8% ± 0.4% and 44.9% ± 3.1%; untreated cells set as 100%). However, Fer‐1 offered little protection at 30 mM SI. Similarly, 6dS treatment showed a protective effect comparable to that of Fer‐1 in a dose‐dependent manner (10–40 μM 6dS; Figure 1A,B). AnxV staining has been used to detect SI‐induced phosphatidylserine (PS) externalisation [20]. Flow cytometric analysis showed that AnxV‐positive cells increased in a SI dose‐dependent manner (10 ~ 30 mM; Figure 1C). Cells pretreated with Fer‐1 and 6dS could reduce the level of AnxV‐positive cells induced by 20 mM SI (13.2% ± 2.8% and 15.4% ± 4.6% versus 43.7% ± 3.3%), but had no such effect under 30 mM SI stimulation (our unpublished data). Ferroptosis is characterised by the accumulation of intracellular ferrous ions (Fe^2+^). Fer‐1 reduces the formation of labile iron pools (LIP), thereby preventing ferroptosis [21]. Solvent/SI‐treated ARPE‐19 cells stained with FerroOrange exhibited intense orange fluorescence, indicating elevated LIP after 16 h of SI stimulation (Figure 1D). The orange fluorescence intensity of cells treated with Fer‐1 and 6dS was reduced approximately 3‐fold compared with 20 mM SI stimulation, showing that 6dS treatment can prevent SI‐induced LIP formation. The control peptide did not affect the SI effects, and the PEDFR inhibitor atglistatin pretreatment abolished the inhibitory effect of 6dS on SI‐induced LIP and cytotoxicity. These findings suggest that 6dS, comparable to Fer‐1, can alleviate SI‐induced ferroptosis in ARPE‐19 cells.

### 3.2 6dS Induces the Expression of SLC7A11, GPX4, and FTH1 in RPE Cells

**FIGURE 2:**
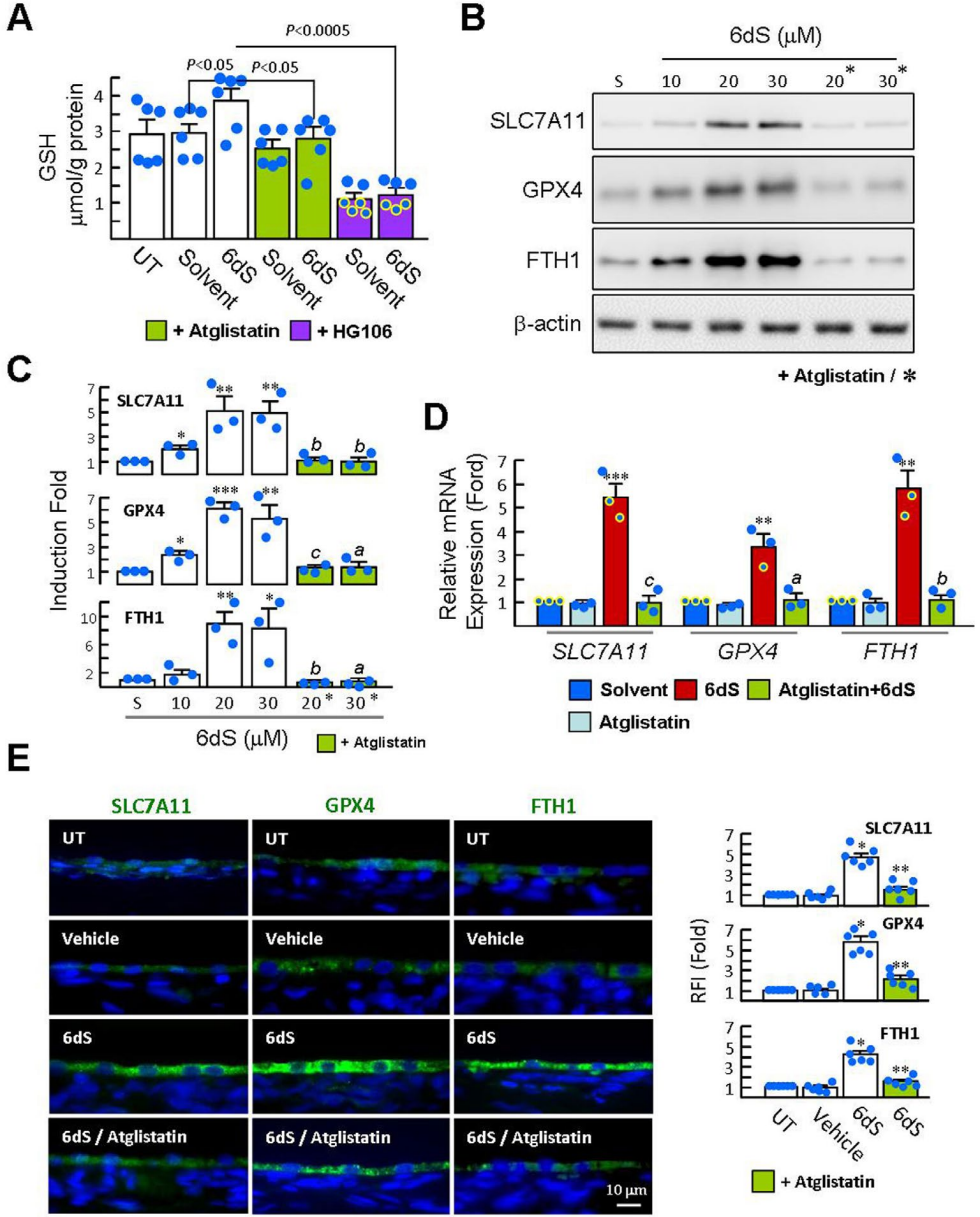
6dS Upregulates GSH levels and negative regulators of ferroptosis in ARPE‐19 cells. (A) ARPE‐19 cells were pretreated with 20 μM atglistatin or 10 μM HG106 for 20 min and then treated with 20 μM 6dS for 6 h, and the reduced GSH levels were measured (*n* = 6). (B and C) Cells were pretreated with atglistatin for 20 min and then treated with 6dS (10–30 μM) for 4 h. The levels of SLC7A11, GPX4 and FTH1 were determined by western blotting. Representative blots and densitometric analyses are from three independent experiments and normalised to β‐Actin. (D) Real‐time qPCR analysis was performed 3 h after 20 μM 6dS treatment. Data are from three independent experiments and normalised to *Gapdh*. *p < 0.05, **p < 0.01, and ***p < 0.001 versus solvent‐treated cells. ^a^ *p* < 0.05, ^b^ *p* < 0.01, and ^c^ *p* < 0.001 versus 6dS‐treated cells. (E) Rats received topical 6dS eye drop (1 mM; 15 μL) four times over 1 h, each administered 15 min apart. Vehicle‐treated rats served as controls. Atglistatin was administered via subconjunctival injection prior to 6dS treatment. After treatment with 6dS eye drops for 6 h, immunofluorescence staining of SLC7A11, GPX4 and FTH1 in RPE cells was performed. Representative images and corresponding RFI (*n* = 6 per group) are from three independent experiments (*n* = 6 per group). Nuclei were visualised using Hoechst 33258 stain (blue). Arrows indicate the RPE cell layer. Data are expressed as mean ± SEM. *p < 0.0001 versus vehicle group. **p < 0.0005 versus 6dS group. RPE, Retinal pigment epithelium.

GSH depletion is known to induce ferroptosis in RPE cells [14]. We investigated the potential mechanism of ferroptosis negative regulation (FNR) induced by 6dS and found that 6dS treatment for 6 h significantly increased GSH levels compared with the solvent control (Figure 2A). In addition, after ARPE‐19 cells were treated with 6dS (10 ~ 30 μM) for 4 h, western blot analysis showed approximately 2‐ to 5‐fold increases in SLC7A11 protein levels compared to the solvent control (Figure 2B,C), supporting that 6dS activates GSH synthesis. Similarly, pretreatment of cells with the SLC7A11 inhibitor HG106 not only reduced the amounts of intracellular GSH content but also blocked the ability of 6dS to stimulate GSH synthesis (Figure 2A). In addition, western blotting showed that 6dS induced the expression of GPX4 and FTH‐1 in a dose‐dependent manner. Real‐time qPCR further showed that 6dS could upregulate the expression of *SLC7A11*, *GPX4 and FTH1* genes (Figure 2D).

Our recent study showed that 6dS could diffuse from the ocular surface into the retinal tissue and RPE layer 1 h after topical application of 6dS eye drops (2 μg/15 μL; 0.2 mM) in rat eyes [10]. Immunofluorescence staining showed increased SLC7A11, GPX4 and FTH1 expressions in the RPE layer 4 h after topical 6dS treatment, compared with vehicle‐treated rats (Figure 2E). Notably, the ability of 6dS to induce the SLC7A11/GSH/GPX4 axis and FTH1 was abolished by atglistatin in vitro and in rats, suggesting that one of the effects of PEDFR signalling is related to FNR.

### 3.3 RPE Cells Pretreated With 6dS Reduces the Consumption of Ferroptosis Negative Regulators Under SI Challenge

**FIGURE 3:**
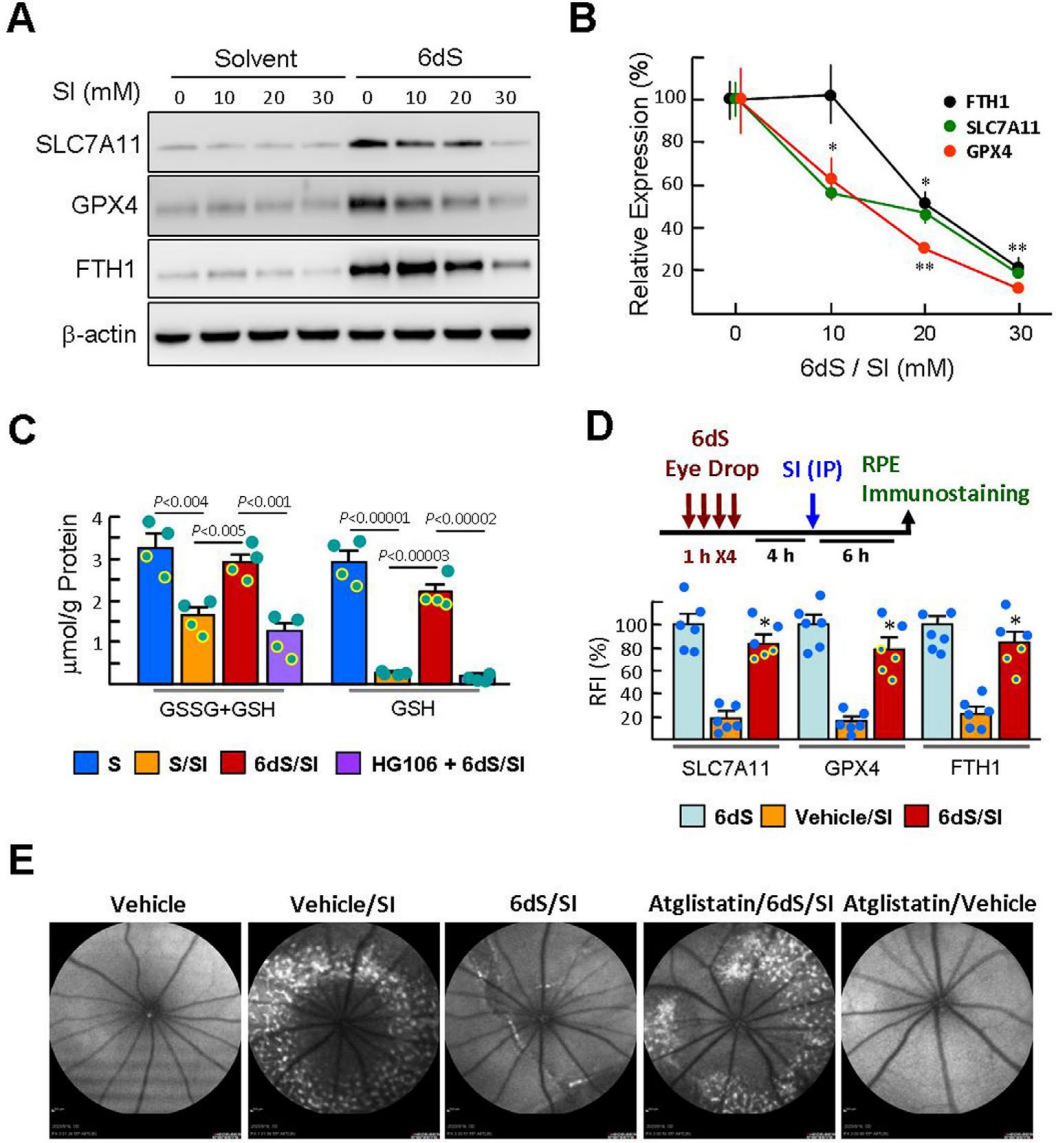
6dS Reduces SI‐induced fundus accumulation of photoreceptor debris, consistent with partial retention of ferroptosis negative regulators in RPE cells. (A and B) ARPE‐19 cells were pretreated with DMSO solvent or 6dS for 4 h and then exposed to SI (10–30 mM) for 3 h. The levels of SLC7A11, GPX4 and FTH1 were determined by western blotting. Representative blots and densitometric analyses are from three independent experiments and normalised to β‐Actin. *p < 0.05 and **p < 0.01 versus 6dS‐treated cells. (C) Cell processing was performed as described above. Levels of total GSH and reduced GSH were determined from four independent experiments. (D and E) Experimental schema: Sd rats were treated topically with 6dS for 4 h as described in the legend of Figure 2E Subsequently, rats were injected intraperitoneally by SI. Immunofluorescent staining of the RPE layer (*n* = 6) and fundus examination (*n* = 3, similar results) were performed 6 h and 7 days after SI injection. *p < 0.001 versus vehicle/SI‐treated cells.

We further performed western blotting to detect the levels of SLC7A11, GPX, and FTH1 after 3 h of SI (10, 20 and 30 mM) stimulation. As shown in Figure 3A, these 6dS‐upregulated proteins decreased after SI stimulation and dropped sharply after 30 mM SI challenge. In contrast, SLC7A11, GPX4 and FTH1 were less sensitive to 20 mM SI challenge (46.8% ± 8.2%, 29.7% ± 5.3% and 51.4% ± 10.2%; cells treated with 6dS alone set as 100%; Figure 3B). Furthermore, 20 mM SI stimulation caused a decrease in total GSH (GSSG + GSH) and reduced GSH levels to 50% and 9%, respectively, compared with solvent‐treated control cells (Figure 3C), whereas 6dS/SI‐treated cells retained 89% and 75%, respectively. HG106 pretreatment abolished the effect of 6dS in alleviating SI‐induced GSH depletion, supporting a higher steady‐state level of GSH in 6dS‐treated cells (Figure 2A).

In SD rats that received topical 6dS eye drops and SI injection for 6 h (6dS/SI group), SLC7A11, GPX4 and FTH1 immunostaining in the RPE layer showed obvious signals comparable to those in the 6dS‐treated group and were approximately 4~5‐fold higher signal intensity than the vehicle/SI group (Figure 3D), suggesting that the 6dS‐induced FNR is still effective under SI challenge. Injection of SI in animals results in primary RPE failure followed by progressive retinal degeneration over time, including the formation of autofluorescence spots in the retinal fundus that are associated with the accumulation of photoreceptor debris [22]. Seven days after SI injection, fundus examination revealed a large number of autofluorescence spots in the vehicle/SI group compared with the vehicle control and 6dS/SI groups (Figure 3E). Injection of atglistatin abolished the protective effect of 6dS on the retina, consistent with its elimination of 6dS‐mediated FNR in RPE cells.

### 3.4 6dS Diminishes SI‐Induced Lipid Peroxidation by Inducing the SLC7A11/GSH/GPX4 Axis in RPE Cells

**FIGURE 4:**
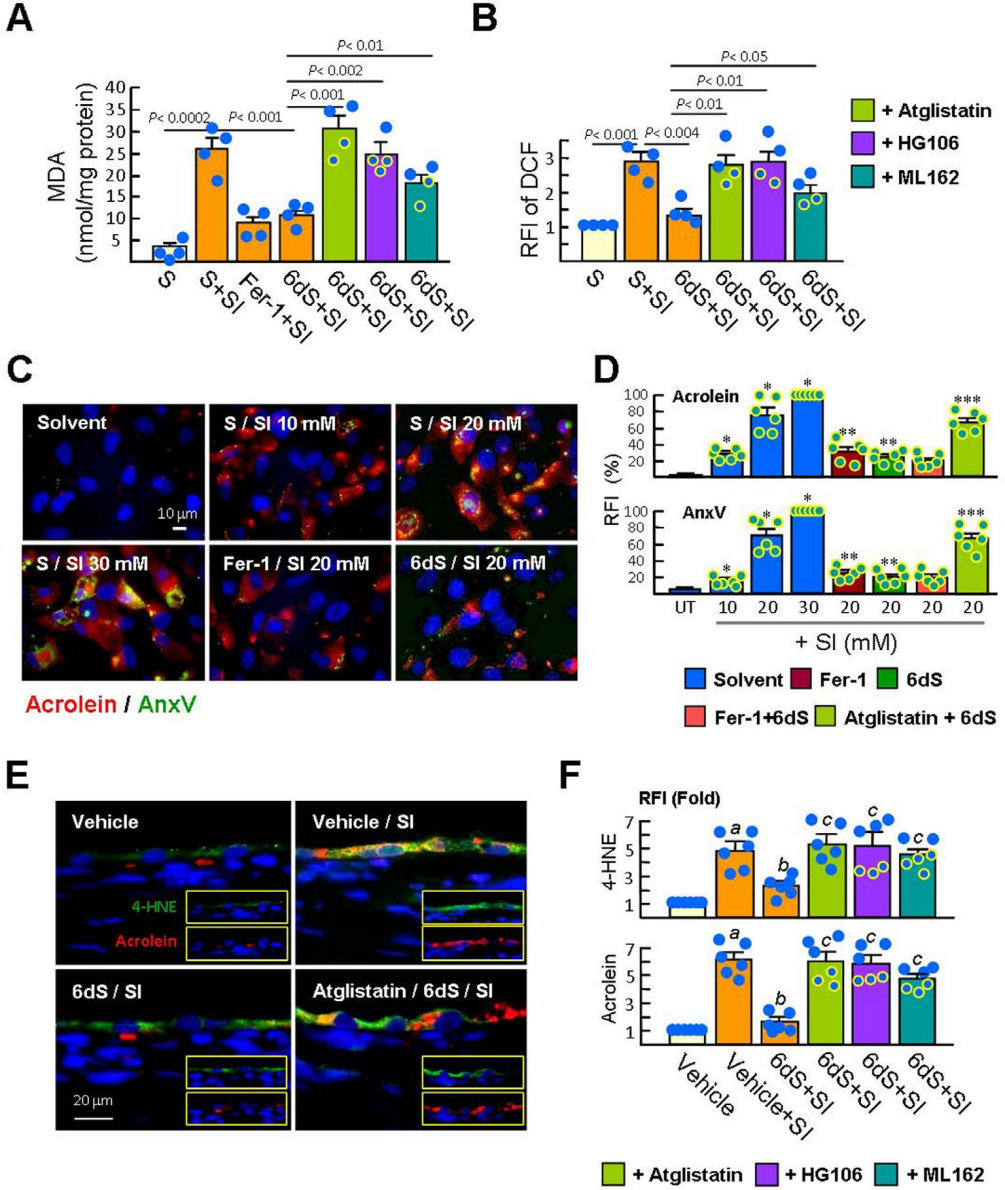
6dS reduces SI‐induced lipid peroxidation in RPE cells. (A and B) The experimental procedures are described in the legend of Figure 1. The levels of MDA and ROS were measured 16 h and 4 h post‐SI challenge (*n* = 4), respectively. (C and D) Membrane disruption and lipid peroxidation were detected by FITC‐AnxV staining and acrolein immunofluorescent staining (red), respectively, as described in Methods. Representative images and RFI of AnxV and acrolein in differently treated cells are shown (*n* = 6). Data are expressed as mean ± SEM. *p < 0.001 versus untreated cells. **p < 0.005 versus 20 mM SI‐treated cells. ***p < 0.00001 versus 6dS/SI‐treated cells. (E and F) Treatment with 6dS eye drops was performed as described in Figure 3D. Double immunofluorescence staining of 4‐HNE (green) and acrolein (red) in the RPE layer of rats 21 h after SI injection. Representative images and RFI of 4‐HNE and acrolein in each group are shown (*n* = 6). ^a^ *p* < 0.001 versus vehicle group. ^b^ *p* < 0.01 versus vehicle/SI group. ^c^ *p* < 0.05 versus 6dS/SI group.

Activation of GPX4 by GSH is critical for limiting the accumulation of lipid peroxides, thereby maintaining cell membrane integrity [17, 18]. Accordingly, SI (20 mM) significantly increased the levels of intracellular MDA (products of lipid peroxidation; Figure 4A), but this SI effect was significantly prevented by cells pretreated with 6dS or Fer‐1. The formation of lipid peroxides was consistent with the elevation of ROS levels after SI stimulation for 16 h (Figure 4B). The inhibitory effects of 6dS on SI‐induced MDA and ROS were significantly blocked by atglistatin, HG106 and ML162 (GPX4 inhibitor), respectively. Immunofluorescence staining of acrolein, a byproduct of lipid peroxidation, [23] revealed that acrolein fluorescence intensity was correlated with increasing SI doses (30 mM SI set as 100%, Figure 4C,D). Pretreatment with Fer‐1 and 6dS reduced acrolein formation in cells under 20 mM SI challenge (30.5% ± 5.2% and 24.5% ± 3.1% versus 76.0% ± 7.9%). We noticed that almost all acrolein‐positive signals merged with AnxV staining (Figure 4C). Our results suggest that SI‐induced lipid peroxidation leads to exposure of the intracellular membrane compound PS, which is then captured by AnxV. Furthermore, the high staining intensity of acrolein and AnxV in SI‐treated ARPE‐19 cells was further reduced when cells were treated with a combination of Fer‐1 and 6dS, although this trend was not statistically significant compared with cells pretreated with either Fer‐1 or 6dS. In addition, the inhibitory effects of 6dS on SI‐induced acrolein production and AnxV labeling in ARPE‐19 cells were significantly abolished by atglistatin.

In SD rats that received SI injection for 21 h, immunostaining for lipid peroxide markers showed that the RPE layer was strongly stained with 4‐hydroxynonenal (4‐HNE) and acrolein compared with the control group (4.8 ± 0.7 and 6.1 ± 0.5 times higher; Figure 4E,F). Rats pretreated with 6dS eye drops significantly reduced the effect of SI on the RPE layer. However, subconjunctival injection of atglistatin, HG106 and ML162 prior to treatment with 6dS eye drops abrogated the 6dS anti‐oxidative effect on RPE cells. Collectively, the results support the involvement of the SLC7A11/GSH/GPX4 axis in 6dS/PEDFR signalling‐induced FNR in RPE cells.

### 3.5 Topical 6dS Eye Drops Prevent SI‐Induced RPE Cell Death in Rats

**FIGURE 5:**
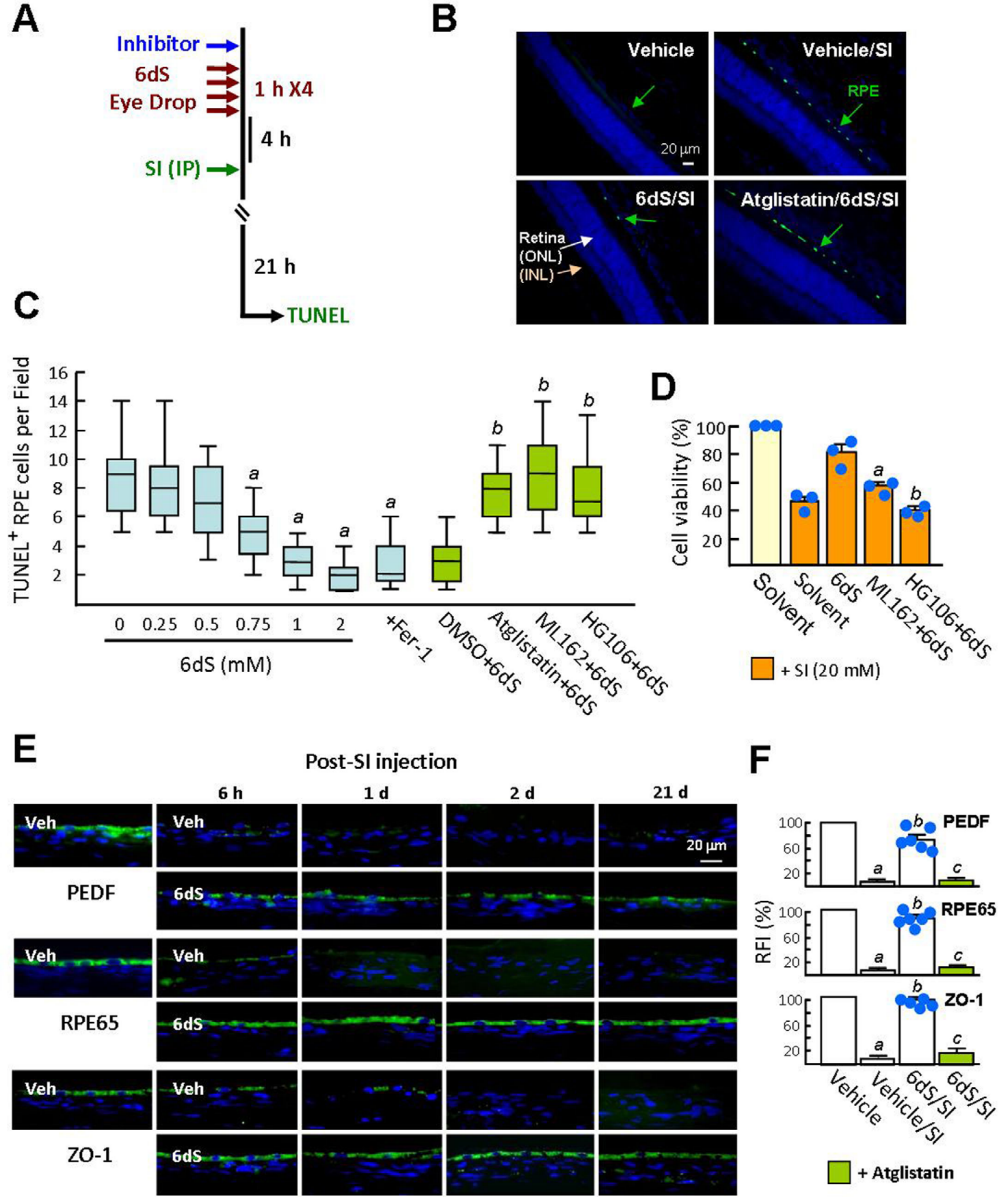
Topical 6dS eye drops reduce SI‐induced RPE cell death and dysfunction in rats. (A) Experimental schema: Treatment with inhibitors and 6dS eye drops was performed as described in the legend of Figure 2E, prior to SI injection. (B and C) Representative images of TUNEL‐positive RPE cells (*green*) in different groups 21 h after SI injection. Data show medians and interquartile ranges (*n* = 6). ^a^ *p* < 0.0005 versus vehicle/SI group, ^b^ *p* < 0.00001 versus DMSO+6dS/SI group. ONL, Outer nuclear layer of the retina. (D) Cell viability was measured by CCK‐8 assay as described in the legend of Figure 1 (*n* = 3). ^a^ *p* < 0.02 and ^*b*^ *p* < 0.003 versus 6dS/SI‐treated cells. (E and F) Treatment with 6dS eye drops was performed as described in Figure 3D. Immunofluorescence staining of PEDF, RPE65, and ZO‐1 in the RPE layer at different time points: 6 h, 1 day, 2 days, and 21 days after SI injection. Representative images and RFI from day 2 after SI injection are shown (*n* = 3). ^a^ *p* < 0.00001 versus vehicle group. ^b^ *p* < 0.0001 versus vehicle/SI group. ^c^ *p* < 0.005 versus 6dS/SI group.

Next, TUNEL assays showed that SI injection for 21 h was able to induce DNA damage in RPE cells (*green colour*; Figure 5A,B). Treatment with 6dS eye drops dose‐dependently prevented the SI cytotoxicity (0.75 ~ 2 mM; Figure 5C). Subconjunctival injection of Fer‐1 reduced the number of TUNEL‐positive cells induced by SI, indicating that ferroptosis plays a key role in SI‐induced RPE cell injury. After subconjunctival injection of the inhibitor solvent (DMSO), topical treatment with 6dS eye drops still blocked RPE DNA damage induced by SI. However, subconjunctival injection of atglistatin, ML162 and HG106 respectively eliminated the antagonistic effect of 6dS eye drops on SI. Similarly, CCK‐8 assay showed that ML162 and HG106 can block the cytoprotective effect of 6dS (Figure 5D). In animals, the expression of RPE markers in the vehicle control group, including retinal pigment epithelial 65 kDa protein (RPE65), zonula occludens (ZO)‐1, and PEDF, was confirmed by immunofluorescence staining (Figure 5E). Their levels dropped sharply 6 h after SI injection and decreased by approximately 90% within 2 days after SI injection, revealing a phenotype of RPE degeneration. 6dS eye drops protected such proteins expressed in RPE cells under SI challenge, but this protective effect was eliminated by atglistatin (Figure 5E,F).

### 3.6 Topical 6dS Eye Drops Alleviate SI‐Induced Retinal Atrophy and Functional Defects in Rats

**FIGURE 6:**
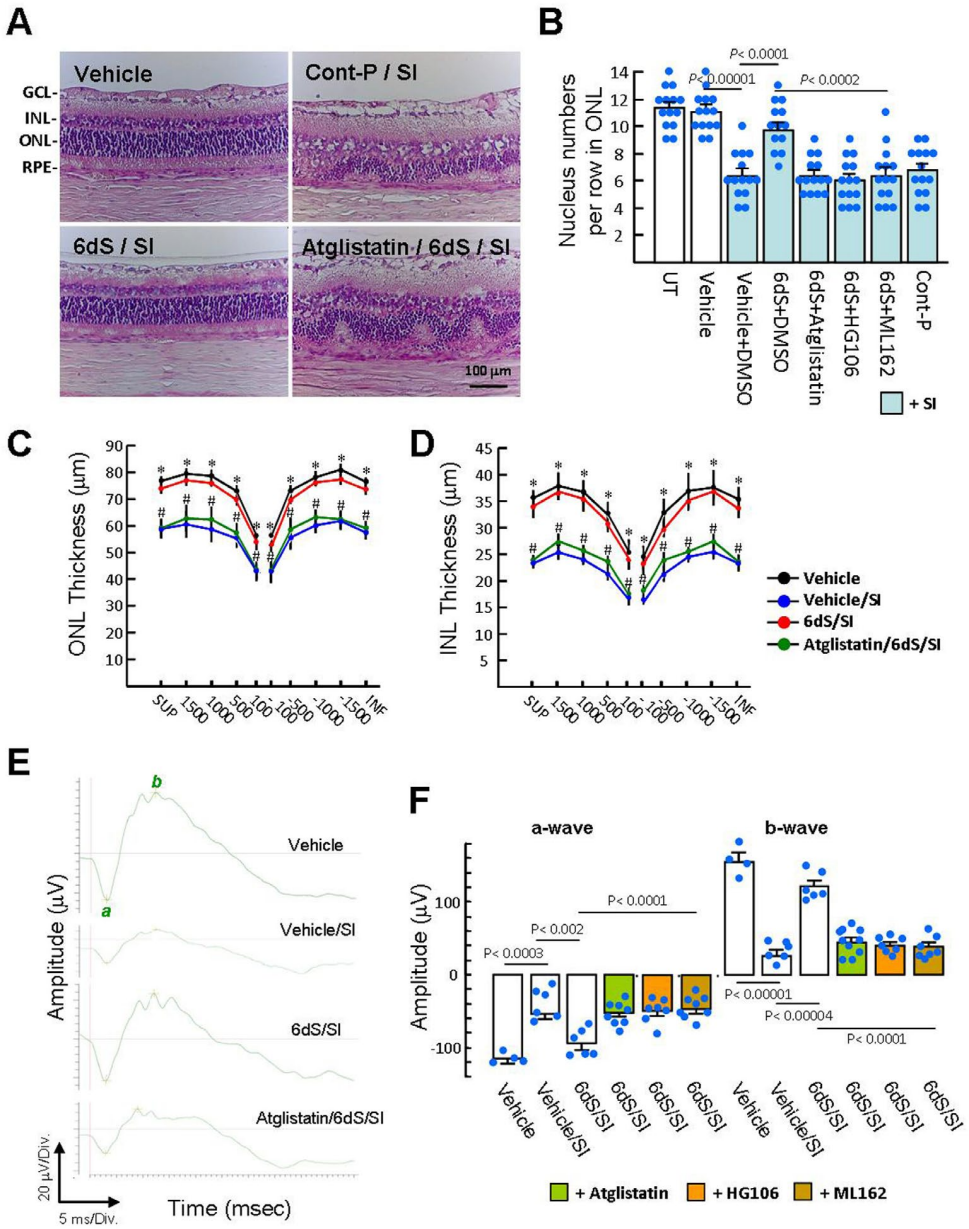
Histological and functional assessment of the effects of 6dS on SI‐induced retinal damage. Treatment with 6dS eye drops was performed as described in Figure 3D. (A–D) Representative photographs of H&E stained retinas 21 days after SI injection. Nuclei/row in the ONL and thickness of the ONL and INL were measured in the inferior (INF) and superior (SUP) retinas as described in Methods. Values are presented as means ± SEM (*n* = 6). *p < 0.01 versus vehicle/SI group. ^#^ *p* < 0.03 versus 6dS/SI group. (E and F) Representative scotopic ERG responses (0 dB; 3.0 cd.s/m^2^) recorded on day 21 post‐SI injection (*n* = 6). GCL, Ganglion cell layer.

The retinal structure is well‐organised, with clearly defined layers. The retinal structure examined using H&E staining 21 days after SI injection showed significant RPE cell loss and retinal atrophy, including reduction in the number of nuclei per row in the ONL, thinning of the INL and ONL, with marked disorganisation in both layers (Figure 6A–D). The 6dS/SI group showed an organised retinal structure similar to the vehicle control, which may be attributed to its effect on RPE protection during early SI challenge. Meanwhile, changes in retinal function were examined through the scotopic ERG. The retina's electrical response to light is generated by photoreceptor cells (a‐wave) and other retinal cells such as Müller cells and bipolar cells (b‐wave; Figure 6E). SI treatment attenuated ERG responses characterised by substantially reduced a‐wave and b‐wave amplitudes compared with vehicle controls (53.8 ± 6.9 and 26.0 ± 6.8 μV versus 115.5 ± 4.2 and 154.5 ± 10.4 μV; Figure 6F), whereas the 6dS/SI group yielded obvious a‐ and b‐wave amplitudes (94.0 ± 7.5 and 120.7 ± 8.0 μV). The protective effects of 6dS on retinal morphology and function were abolished by atglistatin, HG106 and ML162, respectively, reflecting that 6dS activity is closely related to the PEDFR‐induced FNR.

## 4 Discussion

In this study, the 6dS peptide, similar to the ferroptosis inhibitor Fer‐1, effectively abrogated SI‐induced toxicity in ARPE‐19 cells. SI stimulation exhibited hallmark features of ferroptosis, including labile iron accumulation, lipid peroxidation with cell membrane rupture, ROS formation, and GSH depletion as previously described [13, 24, 25]. We provide evidence for the first time that 6dS can induce SLC7A11 expression, thereby increasing GSH levels to alleviate SI‐induced intracellular redox imbalance. Experimental animals injected with SI showed RPE degeneration and retinal atrophy, recapitulating key features seen in dry AMD patients [13, 25]. Topical treatment with 6dS eye drops protected RPE and retina under SI challenge, accompanied by a significant expression of PEDF in RPE cells. Retinal PEDF supported by functional RPE may help maintain retinal health. 6dS also induced the expression of GPX4 and FTH1 in RPE cells, supporting previously reported PEDF effects [15]. We further found that the protective effect of 6dS on SI‐induced RPE ferroptosis in vitro and in vivo was blocked by inhibitors targeting PEDFR, SLC7A11, and GPX4. Therefore, the upregulation of the SLC7A11/GSH/GPX4 axis in RPE cells may be one of the PEDF‐mediated antioxidant mechanisms to counteract the pathogenesis of oxidative stress‐induced RPE damage and retinal degeneration.

Dry AMD is the leading cause of visual impairment in older adults, and patients have limited treatment options. PEDF is considered a promising drug for the treatment of such retinal degenerative diseases due to its antioxidant activity. For example, PEDF protects human RPE cells from H_2_O_2_‐induced damage by promoting GSH synthesis [26]. PEDF is known to increase GPX and GSH levels in retinal pericytes, thereby alleviating oxidative stress damage caused by H_2_O_2_ and high glucose [27]. In this study, we propose that the SLC7A11/GSH/GPX4 axis serves as a critical defence mechanism in 6dS‐treated RPE cells to scavenge excess ROS and lipid peroxides induced by SI. In addition to RPE cells, whether PEDF/6dS activates the antioxidative response in other types of retinal cells remains to be investigated.

SI injections in experimental animals have been widely used to study the impact of oxidative stress‐induced RPE damage on the pathogenesis of dry AMD [22, 28]. After high‐dose SI injection (40 mg/kg), TUNEL assays have been reported to show that significant damage can be observed in RPE cells as early as day 1, with adjacent photoreceptor damage emerging as early as day 3 [29]. Our study showed similar observations in the RPE layer, with evidence of lipid peroxidation in RPE cells. Fer‐1 pretreatment effectively prevents SI‐induced RPE cell death, supporting the notion that ferroptosis is the primary mechanism of this process in vivo [25, 30]. Numerous studies have highlighted that multiple cell death mechanisms, including apoptosis, necroptosis, pyroptosis and ferroptosis, are involved in RPE degeneration and atrophy under different stress conditions associated with dry AMD [31, 32]. Interestingly, we observed a dramatic increase in 4‐HNE levels in RPE cells after SI injection. 4‐HNE has been shown to activate ferroptosis and necroptosis pathways in ARPE‐19 cells [32]. Further studies are needed to explore the protective function of 6dS eye drops against various oxidative insults in RPE cells.

In this study, we found that 6dS eye drops had similar effects compared with PEDF and fer‐1 in suppressing RPE death in SI‐induced dry AMD model [15, 25, 30]. Our finding may provide a new way to protect RPE cells and delay the development of dry AMD. In our current study, the detailed mechanism of FTH1‐mediated FNR under cell preconditioning with 6dS/PEDFR signalling is still lacking. SI‐induced Fe^2+^ accumulation may partially bind to ferritin (a protein composed of FTH1 and ferritin light chain), thereby blocking the formation of LIP and Fe^2+^‐dependent ferroptosis. Additionally, overexpression of FTH1 is able to reduce ferritin degradation caused by NCOA4 (nuclear receptor coactivator 4) cargo receptor‐mediated autophagy (ferritophagy), thereby preventing 6‐hydroxydopamine induced ferroptosis in PC‐12 cells [33]. Whether the FNR effect of 6dS involves inhibition of ferritinophagy via FTH1 induction or NCOA4 downregulation will be investigated in future studies.

In summary, our results confirm that SI is a well‐established oxidant for studying ferroptosis‐related RPE degeneration in animals and that PEDF acts as a negative regulator of ferroptosis in RPE cells. We report that 6dS eye drops can ameliorate SI‐induced RPE and retinal degeneration in rats. Mechanistically, 6dS induces the expression of SLC7A11, GPX4, and FTH1 in RPE cells. Therefore, our study proposes an antioxidant mechanism whereby PEDF‐R signalling is linked to system Xc^−^ and participates in GSH metabolism to counteract intracellular redox imbalance. Further exploration of the direct interaction of 6dS and PEDF‐R may provide a more complete understanding of 6dS pharmacology and further support the concept that endogenous PEDF acts on FNR. Our findings highlight that activation of PEDF‐R in RPE cells may be an effective strategy to prevent retinal degeneration‐related diseases, including dry AMD.

## Author Contributions

**Tsung‐Chuan Ho:** conceptualization (equal), data curation (equal), formal analysis (equal), investigation (equal), writing – original draft (equal). **Shawn‐H Tsai:** data curation (equal), methodology (equal), project administration (lead), supervision (supporting), validation (lead), visualization (lead). **Shu‐I Yeh:** data curation (lead), formal analysis (lead), funding acquisition (supporting), investigation (supporting), supervision (lead), validation (supporting), visualization (supporting). **Ming‐Hui Sun:** data curation (equal), formal analysis (equal), investigation (supporting), methodology (supporting), software (equal), visualization (supporting). **Yeou‐Ping Tsao:** conceptualization (equal), funding acquisition (equal), methodology (equal), supervision (equal), validation (equal), writing – review and editing (equal).

## Conflicts of Interest

The authors declare no conflicts of interest.

## Data Availability

The datasets used and/or analysed during the current study are available from the corresponding author upon reasonable request.
